# Determinants of evidence use by frontline maternal, newborn and child health staff in selected health facilities in Ghana

**DOI:** 10.1186/s12961-022-00881-8

**Published:** 2022-06-28

**Authors:** Gordon Abekah-Nkrumah, Doris Ottie-Boakye, Johnson Ermel, Sombié Issiaka

**Affiliations:** 1 Department of Public Administration and Health Services Management, University of Ghana Business School, Legon, P. O. Box 72, Accra, Ghana; 2 Regional Institute for Population Studies, University of Ghana, Legon, P. O. Box LG 96, Accra, Ghana; 3 West African Health Organisation, 01 BP 153 Bobo-Dioulasso 01, Burkina Faso

**Keywords:** Evidence, Frontline health workers, Maternal and Child Health, Practice decisions, Ghana

## Abstract

**Background:**

The current paper examines the level of use of evidence and factors affecting the use of evidence by frontline maternal, newborn and child health (MNCH) and reproductive and child health (RCH) staff in practice decisions in selected health facilities in Ghana.

**Methods:**

Data on use of evidence and its correlates was collected from 509 frontline healthcare staff drawn from 44 health facilities in three regions in Ghana. Means were used to examine the level of use of evidence, whiles cross-tabulations and Partial Least Squares-based regression were used to examine factors associated with the use of evidence in practice decisions by frontline MNCH/RCH staff.

**Findings:**

The findings suggest a high level of use of evidence by frontline MNCH/RCH staff in practice decisions (score of 3.98 out of 5), albeit that evidence use is skewed towards the use of practice guidelines and policies. For the antecedents of evidence use, attitude had the highest score (3.99), followed by knowledge (3.8), access to evidence (3.77) and organizational structure (3.57), using a threshold of 5. The regression results indicate that attitudes and knowledge of frontline MNCH/RCH staff, organizational structure (strongest association), years of experience, being a male and working in a mission health facility are significantly positive correlated with evidence use, whiles working in a private health facility or in the post-natal clinic is negatively correlated with the use of evidence.

**Conclusion:**

We argue that any effort to improve the use of evidence by frontline MNCH/RCH staff in practice decisions should focus on improving attitudes and knowledge of staff as well as challenges related to the structure of the organisation. Given however that the score for attitude was relatively high, emphases to improve evidence use should be on access to evidence and organizational structure in particular, which had the lowest score even though it has the strongest association with the use of evidence.

**Supplementary Information:**

The online version contains supplementary material available at 10.1186/s12961-022-00881-8.

## Introduction

The health of mothers, children and infants have been prioritized by both international and national organizations. Per Millennium Development Goal (MDGs) 4 and 5, Under-5 and maternal mortality were expected to be reduced by two-thirds and three-quarters respectively by 2015 [1]. Several developing countries and particularly, those in the Sub-Saharan African (SSA) region, including Ghana, could not meet these targets [1]. Consequently, the Sustainable Development Goals (SDGs), the “successor” of the Millennium Development Goal (MDGs) seeks to improve maternal and child health by reducing neonatal mortality to at least 12 per 1000 live births and under-5 mortality to 25/1000 live births by 2030 [1]. Similarly, maternal mortality is expected to reduce to less than 70/100,000 live births during the same year [1].

Notwithstanding the above, reproductive and child health (RCH) outcomes in Ghana have improved substantially over the last two decades. Nationally, the maternal mortality rate (MMR) reduced by 59.2% from 760/100,000 live births in 1990 to 310/100,000 live births in 2017 [2, 3]. Other reproductive health inputs have also seen improved consumption; with 4 + antenatal visits reported to be 89% and women aged 15–49 years receiving antenatal care (ANC) from a skilled provider being 98% [3]. In addition, neonatal tetanus vaccination coverage for women stood at 78% in 2014, while delivery in a health facility improved to 79% in 2017 from 42% in 1988 and assisted deliveries from 44% in 1988 to 80% in 2017 [3, 4]. Additionally, 84% of women received postnatal check-up within the first two days after delivery in 2017 [3].

In terms of infant and child health, similar gains have been recorded over the last two and half decades. There has been 61% increase in basic vaccination coverage between 1988 (47%) and 2014 (77%) [4]. As at 2014, the neonatal mortality rate was reported to be 29/1000 live births while perinatal mortality was 38/1000 live births. Again, under-5 (155/1000), infant (77/1000) and neonatal (43/1000) mortality in 1988 has reduced to 52/1000, 37/1000 and 25/1000 respectively in 2017 [3].

Notwithstanding these improvements, Ghana did not meet MDG 4 and 5. Besides, quality of care in many Ghanaian health facilities, especially in maternal, newborn and child health (MNCH) and RCH remains a challenge [5]. It is in the context of dealing with these challenges that the use of evidence to support decision-making, especially at the clinical level becomes important. Consequently, evidence products such as health policies, guidelines, protocols as well as evidence from academic and facility-based operations research have become an important part of the knowledge base for service delivery at every level. These instruments are designed mostly to improve quality of care, reduce variation in practice and ensure that evidence-based care is delivered to clients [6]. As it is the case in other countries, Ghana uses both internationally and nationally developed guidelines for the purposes of standardizing and improving quality of care. A typical reference point is MNCH or RCH, where several policies, protocols and guidelines have been developed to help improve quality of care and standardize MNCH/RCH care delivery. It is important to emphasise that MNCH/RCH related policies, protocols and guidelines within the Ghana Health Service (GHS) and the Ministry of Health (MoH) are based on existing scientific evidence [5].

Notwithstanding potential benefits associated with the use of these instruments, evidence in the literature suggest that they are hardly used by frontline health workers, thereby limiting their potential for realizing associated benefits [7–9] such as improving quality of care [8, 10]. Moreover, existing guidelines and protocols both at the national and international levels are believed to be underutilized, because frontline health workers fail to adopt them, hence, their limited impact [11–14]. For example, the results of a study that includes Burkina Faso, Tanzania and Ghana suggest that maternal health guidelines in these countries were found to be of good quality in content, yet usage by frontline healthcare workers was limited [15]. Additionally, evidence from the Greater Accra [16] and Ashanti [17] regions of Ghana suggest that there is minimal use of existing evidence in public health practice decisions by frontline health workers. The discussion above suggests that the use of various forms of evidence to inform health-related decisions has generally attracted substantial attention both in the academic literature and policy discourse in Ghana. However, the extent to which evidence is used in making MNCH/RCH decisions by frontline health workers has received limited attention. Beside the cross-country analysis that included Ghana [15], there is currently only one published paper on Ghana [5] that focused specifically on the use of evidence to inform MNCH/RCH decisions. This paper however, focused mainly on the use of evidence to inform MNCH/RCH policy (macro level) and not decision-making by frontline clinical staff to aid practice decisions.

The current paper therefore examines the level of use of evidence (i.e. health policies, guidelines, protocols as well as evidence from academic and facility-based operations research etc.) by frontline MNCH/RCH staff in making practice decisions. Specifically, the paper.

1. Examines the level of use of evidence by frontline MNCH/RCH staff in making practice decisions in selected health facilities in Ghana
2. Examine factors that influence the use of evidence in practice decision-making by frontline MNCH/RCH staff in selected health facilities in Ghana.

Although it is not standard for frontline health staff to synthesize evidence from academic research findings for purposes of decision making, it is common for higher end health facilities to synthesize research findings to inform team decisions or also use the findings of facility conducted operations research to inform decisions. Thus, evidence in the context of this study is operationalized to include evidence products such as health policies, guidelines, protocols as well as use of evidence from academic and facility-based operations research.

## Methodology

### Study design

The study is based on a cross-sectional design with health facilities carefully selected to include those in rural and urban areas as well as areas that do not have demographic surveillance sites. Demographic surveillance sites were excluded from the study sample to ensure that on-going interventions in those sites do not confound/interfere with the findings of the current study.

### Sampling method

The study targeted frontline health workers (medical doctors, nurses, midwives, public health officers, enrolled nurses, registered general nurses, and community health nurses) in the Greater Accra, Ashanti and Eastern regions of Ghana, who are involved in the provision of MNCH/RCH services. The choice of the 3 regions is on the basis that they have the highest number and types (i.e. by ownership and hierarchy) of health facilities. Having the highest number of health facilities was important for having an adequate sample size for the study, whiles diversity in ownership and hierarchy was essential for selecting health facilities that are representative of the population of health facilities in Ghana.

Health facilities for the study were conveniently selected from the three regions based on accessibility and willingness to participate in the study. The selection was however done in a way to account for diversity in the types (ownership and hierarchy) of health facilities in a region. The health facilities selected include Ashanti: 8 Community Health and Planning Services (CHPS) Compounds, 6 Health Centers, 3 Maternity Homes/Clinics, 4 Private Hospitals, 2 Mission Hospitals, 2 District Hospitals, 1 Poly Clinic and 1 Regional Hospital. Although a tertiary hospital was originally part of the Ashanti sample, conditions for securing access was almost impossible to achieve and so the tertiary facility was dropped. In Greater Accra, there were 4 CHPS Compounds, 5 Health Centers, 2 Private Hospitals, 1 Mission Hospital, 1 Regional Hospital, 1 Tertiary Hospital and 1 Qusai-Government Hospital. In the Eastern region, 1 Mission Hospital, 1 Regional Hospital and 1 Poly Clinic were selected. The relatively smaller number of health facilities from the Eastern region is based on health facility willingness to participate. Overall, there were 12 CHPS compounds, 11 Health Centers, 3 Maternity Homes/Clinics, 6 Private Hospitals, 3, Mission Hospitals, 2 District Hospitals, 2 Poly Clinics, 3 Regional Hospitals, 1 Quasi Government Hospital and 1 Tertiary Hospital.

After the selection of health facilities, the total number of MNCH/RCH staff in each health facility selected was ascertained and a representative sample calculated using 95% confidence level and 5% margin of error. Proportional representation was then used to distribute the calculated sample across the different cadre of MHNCH/RCH staff (medical doctors, nurses, midwives, public health officers, enrolled nurses, registered general nurses, and community health nurses) in each health facility. However, the selection of staff from each cadre to respond to the survey was done based on availability and willingness to participate due to the shift system operated by frontline MNCH/RCH staff, in addition to the fact that their heavy workload meant it was impossible to interview some of them. Overall, a total of 509 staff from all the health facilities selected completed a questionnaire each administered by enumerators.

### Instrument and data collection

The instrument contained questions on socio-demographic characteristics of respondents (age, sex, marital status, education, years of work and licensure, professional group, unit of assignment etc.), use of evidence, and key factors that influence the use of evidence by frontline MNCH/RCH staff in making practice decisions. Specific questions used to capture the use of evidence and key factors influencing the use of evidence were based on instruments used by prior authors [18–22]. The use of evidence was captured by a 19-item questionnaire that assessed the use of research findings, protocols, practice guidelines, policies etc. by frontline MNCH/RCH staff in practice decision. However, factors that influence the use of evidence by frontline MNCH/RCH staff were assessed using domains on attitudes (8-item), knowledge (14-item), access (7-item) and organisational structure (28-item). For each of the domains listed above, specific questions that capture the main concept of the domain were asked, with the respondent expected to indicate their agreement or not to the statement (Likert’s scale: 1 = strongly disagree; 2 = disagree; 3 = not sure; 4 = agree; 5 = strongly agree). Given that the data collected was self-reported, there could be some level of self-reported bias in the responses. However, the results of a follow up qualitative study (not part of this paper) confirms the current findings. This suggest that the presence of self-reported bias in the current study may be limited. A Copy of the data collection tool has been added to the paper as supplementary material.

Ethical clearance (with ethical clearance number GHS-ERC010/05/18) was sought from the Ethical Review Board of the Ghana Health Service in addition to administrative approval from relevant regional and district Directors of Health Services as well as heads of participating facilities. Prior to the main data collection, the instrument was amended to reflect the results of a pre-testing of the instrument at the MNCH/RCH unit of the University of Ghana Hospital. The instrument was administered by trained data collectors to respondents using a Computer Assisted Personal Interviewing (CAPI) technology. The CAPI was used to ensure that data collected is transmitted directly and stored in a central data repository. This limited the possibility of errors normally associated with manual capture of data.

### Analysis of data

Descriptive statistics based on frequencies and means were used to examine the extent of use of evidence. In addition, an ANOVA test was used to examine differences in the use of evidence between different groups (health facility ownership, location, gender, age group, religious affiliation, marital status, education level, workload, and years of practice). A Partial Least Squares approach via structural equation modeling was used, with the help of version 3 of SmartPLS software to examine factors that influence the use of evidence by frontline MNCH/RCH staff in their practice decisions. In estimating the structural equation, measurement models that account for reliability of the constructs were assessed, starting with convergent validity, which examines the internal consistency of the constructs. This was assessed using the Cronbach Alpha scores, the composite reliability scores and the AVE scores. An internally consistent construct must have a Cronbach Alpha and a composite reliability score to be at least 0.7. Also, indicators for each construct must explain at least 50% of the total variation (AVE score of at least 0.5). Table 1 shows that the retained indicators were internally consistent as each of the construct scored above the threshold value of 0.7.

**Table 1:** Convergent validity assessments

Variable	Cronbach’s alpha	Composite reliability	Average variance extracted (AVE)
Access	0.73	0.83	0.55
Attitude	0.73	0.83	0.56
Evidence	0.91	0.92	0.50
Knowledge	0.91	0.93	0.51
Org structure	0.92	0.93	0.51

*Source* Constructed by authors based on field data

Secondly, discriminant validity assessments were done to examine the ability of an indicator to uniquely describe its own construct, and not another construct. The Fornel–Larcker criterion assesses the inter-construct correlations, against the square root of the AVE scores of each of the construct. Discriminant validity is established if the square root of the AVE score for a construct is greater than its correlation with other items. Table 2 shows that the Fornel–Larcker criteria were met for discriminant validity.

**Table 2:** Discriminant validity assessments

Variable	Access	Attitude	Use of evidence	Knowledge	Org structure
Access	0.74				
Attitude	0.61	0.75			
Use of evidence	0.49	0.46	0.71		
Knowledge	0.64	0.53	0.68	0.71	
Org structure	0.40	0.34	0.62	0.58	0.71

*Source* Constructed by authors based on field data

Henseler et al. [23] provides a new method for assessing the establishment of discriminant validity on the basis that the Fornel–Larcker criterion do not reliably detect the lack of discriminant validity in reflective latent variables or constructs. The Heterotrait–Monotrait (HTMT) ratio is calculated by examining the ratio of Monotrait–Hetrotrait correlations and Heterotrait–Heteromethod correlations. These are respectively the correlations of indicators measuring the same constructs and cross correlations of indicators measuring different constructs [23]. According to Kline [24], a well-established discriminant validity has a score less than 0.85. Results shown in Table 3 provides evidence of the establishment of discriminant validity based on the HTMT assessments.

**Table 3:** HTMT criterion for discriminant validity assessments

Variable	Access	Attitude	Evidence	Knowledge
Access				
Attitude	0.84			
Evidence	0.6	0.56		
Knowledge	0.79	0.65	0.74	
Org structure	0.49	0.41	0.66	0.63

*Source* Constructed by authors based on field data

## Findings

### Socio-demographic characteristics of respondents

The different health facilities were re-categorized (Private, Government, Mission and Qusai Government) for ease of analysis as in Table 4. Respondents were largely female (94.5%), with majority (66.75%) having a diploma certificate and above in terms of education. As expected, majority (83.1%) of the respondents were from urban health facilities, 83.6% have between 0 to 10 years of practice experience and 93.7% have worked in the current health facility for 10 years and below. About 80% of those interviewed work for less than 8 h a day, with the remaining 20% working for more than 8 h a day. Also, 49% of the respondents take care of less than 20 patients a day, 31% take care of between 20 to 40 patients a day, with the remaining 20% taking care of between 41 to 80 patients a day. Whiles 54.3% of the respondents indicated that they were married or in some form of a union, 92.4% of the respondents identified themselves as Christian. Finally, about 36% of the respondents work in Child Welfare Clinics, 31% in Postnatal Care, followed by Delivery (20%) and Antenatal Care (13%).

**Table 4:** Descriptive statistics of constructs

Variables	Freq	%
Facility ownership		
Private facility	106	20.9
Government facility	330	65.1
Mission facility	25	4.9
Quasi facility	46	9.1
Type of location		
Urban	414	83.1
Rural	84	16.9
Respondent sex		
Male	28	5.5
Female	481	94.5
Marital status of respondent		
Currently married	164	50.0
Living together	14	4.3
Widowed	2	0.6
Divorced	7	2.1
Separated	6	1.8
Never married	125	38.1
Don’t know	10	3.0
Highest education obtained		
Certificate	169	33.3
Diploma	221	43.6
First degree	90	17.8
Masters (MA, Mphil, etc.)	11	2.2
PhD	1	0.2
Other	15	2.9
Religious affiliation		
Christian	462	92.4
Islamic	23	4.6
Traditional/other	15	3.0
Years of practice		
Less than 5 years	228	45.5
5–10 years	191	38.1
11–15 years	35	7.0
16–20 years	17	3.4
Above 20 years	30	6.0
Years spent at facility		
Less than 5 years	376	73.4
5–10 years	104	20.3
11–15 years	20	3.9
16–20 years	9	1.8
Above 20 years	3	0.6
Patient load		
Less than 20 patients	250	48.8
20 to 40 patients	160	31.3
41–60 patients	71	13.9
61–80 patients	11	2.1
More than 80 patients	20	3.9
Years of licensure		
Less than 5 years	231	47.8
5–10 years	177	36.6
11–15 years	28	5.8
16–20 years	19	3.9
Above 20 years	28	5.8
Patient work hours per day		
8 h and less	266	79.9
More than 8 h	67	20.1
Unit assigned to		
ANC	60	13.2
Child Welfare Clinic	162	35.5
Delivery	92	20.2
PNC	142	31.1

*Source* Authors computation based on field data

^a^Non responses are excluded from the percentage determinations

### Extent of use of evidence by frontline MNCH/RCH staff

In this section, we present results on the extent of use of evidence by frontline MNCH/RCH staff as well as factors (attitude, access, knowledge and organizational structure) that are correlated with the use of evidence in MNCH/RCH practice decisions. As indicated in the methodology section, each factor/construct was measured using several questions. However, only those indicators with factor loadings of 0.5 and above through a confirmatory factor analysis were retained. Table 5 shows the number of initial questions used and number retained for each construct.

**Table 5:** Retained constructs for model

Construct	Number of indicators (initial)	Number of indicators retained
Attitude	8	5
Access	7	5
Knowledge	14	12
Organizational structure	28	14
Use of evidence	19	10

*Source* Authors computation based on field data

Table 6 presents descriptive statistics (individual and average scores) and indicator loadings of the various indicators that were retained for each construct/factor. For the attitude construct, indicators with higher average scores were related to worker’s interest in learning skills necessary to incorporate evidence-based practice (Mean = 4.21, SD = 0.95) and workers perception of the necessity for use of evidence in their practice (Mean = 4.15, SD = 0.94). For the access construct, availability of research evidence (Mean = 3.99, SD = 0.93) and access to information related to the health practice (Mean = 3.81, SD = 0.94) had the highest average scores. In terms of knowledge, indicators with higher scores included confidence of the worker to share information (Mean = 4.11, SD = 0.92) and confidence in disseminating new ideas with colleagues (Mean = 3.96, SD = 0.97). For organizational structure, continuous learning as key to improvement (Mean = 3.82, SD = 0.98) and the facility’s ability to promote a climate of openness, respect and trust for professionals (Mean = 3.7, SD = 1.1) came up as indicators with relatively higher average scores. In terms of use of evidence, the utilization of practice guidelines (Mean = 4.03, SD = 0.87) and use of practice policies had the highest average scores (Mean = 3.87, SD = 8.96).

**Table 6:** Descriptive statistics and factor loadings of retained constructs

Constructs/variables	Mean	SD	Loadings
Attitude			
Use of evidence in practice does not waste time	3.74	1.086	0.628
Prefer to change to new methods than sticking to tried and tested methods	3.76	1.181	0.689
Application of evidence-based practice is necessary in practice	4.15	0.936	0.758
literature and research findings are necessary in day-to-day practice	4.09	0.967	0.742
Interested in learning the skills necessary to incorporated evidence-based practice in daily practice	4.21	0.952	0.733
Overall average	3.99		
Access			
Available research evidence is relevant to practice	3.99	0.929	0.618
Possess sufficient understanding of what constitutes evidence in the practice	3.79	0.926	0.708
Have confidence in findings from research	3.79	0.931	0.777
Have degree of access to information related to practice	3.81	0.940	0.677
Research findings often take into consideration reality of one's practice	3.45	1.077	0.685
Overall average	3.77		
Knowledge			
I have above average in the skills for monitoring and reviewing practice skills	3.65	0.985	0.695
I have above average skills to convert my info needs into a research question	3.46	0.972	0.677
I am aware of major sources of info for which I can extract evidence for the purposes of my practice	3.75	0.965	0.73
I am very much capable of identifying gaps in my professional practice	3.92	0.946	0.676
I have enough knowledge to be able to extract evidence from available sources for my practice	3.73	0.942	0.723
I have enough ability to analyse critically, evidence against set standards for my practice	3.63	0.953	0.627
I have enough ability to determine how valid an evidence material is for my practice	3.72	0.922	0.702
I have enough ability to determine how useful (clinically applicable) an	3.81	0.918	0.764
I have enough ability to apply information to individual cases	3.92	0.875	0.733
I am confident in my ability to share ideas and information with colleagues	4.11	0.915	0.715
I am confident in disseminating new ideas and info with colleagues	3.96	0.965	0.734
I have enough ability to review my own practice	3.95	0.970	0.716
Overall average	3.80		
Organisational structure			
We have a system that promotes external contacts and allows us to learn from other organisations	3.39	1.099	0.68
The environment at my workplace embraces change and support of the use evidence for my practice	3.56	1.064	0.742
The facility promotes a climate of openness, respect and trust among all levels of professionals	3.70	1.099	0.672
The basic values of the Department include continuous learning as a key to improvement in client care	3.82	0.981	0.688
Managers frequently involve staff in important decisions relating to clinical care procedures, protocols and guidelines	3.60	1.102	0.652
Managing knowledge is central to the organisation's strategy	3.53	1.010	0.677
Management clearly communicates key research strategy and priorities	3.43	1.085	0.687
There is widespread support and acceptance of the organisation's mission statement	3.52	1.081	0.717
There is strong professional leadership within the organisation that facilitates research	3.38	1.152	0.691
Professionals are encouraged to question their practice	3.58	1.052	0.659
Problems are discussed openly and analytically to learn from experience and without blame	3.65	2.871	0.695
There are best practice repositories in my organisation, recognising and valuing existing knowledge	3.45	0.987	0.686
Attendance at conferences/presentations that give information on research and exposure to new info is encouraged	3.65	1.092	0.696
Professionals are encouraged to discuss experiences/expertise with colleagues in regulate meetings	3.69	1.039	0.688
Overall average	3.57		
Use of evidence			
I often integrate the evidence I find with my expertise into my practice	3.67	0.925	0.706
I often share this information with colleagues	3.89	0.968	0.688
I actively seek practice guidelines available for my practice	3.96	0.908	0.722
I use practice guidelines in my practice	4.03	0.870	0.743
I am aware that practice guidelines are available online	3.98	0.982	0.671
I use professional literature and research findings in the process of clinical decision making	3.75	0.925	0.636
I use practice protocols, policies and guidelines in my practice	3.98	0.878	0.712
I am able to incorporate patient preferences with practice protocols and guidelines	3.87	0.896	0.722
I actively seek practice guidelines and protocols pertaining to areas of my practice	3.89	0.901	0.709
I am confident in my ability to find relevant literature to answer clinical questions	3.85	0.894	0.705
Overall average	3.89		

*Source* Authors computation based on field data

An examination of the overall mean of each of the constructs suggest that the constructs are significantly different from each other (*F* = 8.95, *p* = 0.00) and therefore key in the estimated model. Overall, the attitude construct had the highest average score, followed by the construct on use of evidence. Organisational structure recorded the least average score, which was followed by the construct on access.

### Relationship between socio-demographic characteristics and key constructs of evidence use

In addition to the strength of each of the key constructs (attitude, access, knowledge, organizational structure and use of evidence) captured in Table 6, the relationship between socio-demographic characteristics and each of the key constructs were examined as per Table 7. The results suggest that MNCH/RCH staff in Mission and Quasi health facilities show significantly better attitude and access. In terms of use of evidence, the results suggest that MNCH/RCH staff in mission hospitals do better, followed by Quasi and Government hospitals, with the differences being significant. For location, the results suggest that for each of the constructs, frontline MNCH/RCH staff in urban areas show a better performance except that the difference was significant only in the case of access. Gender and age differences are insignificant, with those declaring as Christian or traditional/other religion being better when it comes to knowledge. As expected, those with post-graduate education are significantly more predisposed to using evidence in their practice followed by those with diploma/certificate. On the contrary, those with bachelor’s degree are significantly more likely to display a better attitude, whiles those with diploma/certificate are significantly more likely to identify with a stronger organizational structure as an antecedent to the use of evidence in practice decisions. Additionally, those who have between 11 to 15 years of practice tend to have a higher score on all the constructs, with the exception of knowledge, where the highest score came from those with 16 to 20 years of practice. It is important to emphasise that the effect of years of practice was not significant for organizational structure and use of evidence. The results also suggest that those who work for more than 8 h, have significantly higher scores on access, knowledge, organizational structure and use of evidence to inform practice decisions compared to those who work for 8 h or less.

**Table 7:** Cross tabulation of demographic characteristics against attitude, access, knowledge and organizational structure

	Attitude	Access	Knowledge	Structure	Use
Facility ownership					
Private	3.82	3.56	3.67	3.43	3.64
Government	4.03	3.78	3.83	3.61	3.90
Mission	4.06	4.00	3.98	3.46	4.17
Quasi	4.14	4.06	3.90	3.48	3.90
*ANOVA/T test p value*	***0.07***	***0.05***	***0.13***	***0.17***	***0.00***
Type of location					
Rural	3.99	3.75	3.79	3.54	3.85
Urban	4.10	3.90	3.88	3.66	3.93
*ANOVA/T test p value*	***0.22***	***0.07***	***0.28***	***0.17***	***0.30***
Sex					
Male	4.00	3.81	3.77	3.41	3.96
Female	3.99	3.75	3.81	3.57	3.85
*ANOVA/T test p value*	***0.95***	***0.65***	***0.81***	***0.30***	***0.41***
Age group					
20–29	3.94	3.71	3.75	3.51	3.84
30–39	4.03	3.79	3.82	3.60	3.86
40–49	4.02	3.75	3.85	3.48	3.95
50 and above	4.06	3.86	3.96	3.79	4.00
*ANOVA/T test p value*	***0.62***	***0.58***	***0.36***	***0.18***	***0.50***
Religious affiliation					
Christian	4.01	3.77	3.82	3.57	3.88
Islamic	3.75	3.70	3.66	3.30	3.68
Traditional/other	4.07	3.96	4.02	3.93	4.03
No religion	3.67	3.30	3.15	3.50	3.70
*ANOVA/T test p value*	***0.29***	***0.32***	***0.05***	***0.19***	***0.39***
Marital status					
Married	4.17	3.77	4.03	3.59	3.92
Single	4.17	3.93	3.98	3.73	4.01
Widowed	4.00	4.00	4.42	4.04	4.25
Divorced	4.26	3.77	3.82	3.64	3.90
Do not know	4.06	3.79	3.86	3.58	3.93
*ANOVA/T test p value*	***0.65***	***0.34***	***0.22***	***0.45***	***0.55***
Highest education level					
Diploma/Certificate	3.95	3.74	3.82	3.63	3.88
Bachelor’s Degree	4.15	3.84	3.71	3.22	3.76
Postgraduate certification	3.90	3.73	3.92	3.45	3.91
*ANOVA/T test p value*	***0.08***	***0.47***	***0.35***	***0.00***	***0.30***
Years of practice					
Less than 5 years	3.85	3.64	3.73	3.55	3.81
5–10 years	4.09	3.81	3.81	3.54	3.89
11–15 years	4.26	4.01	3.98	3.59	3.93
16–20 year	4.19	3.98	4.14	3.67	4.08
Above 20 years	4.05	3.86	3.93	3.65	3.95
*ANOVA/T test p value*	***0.00***	***0.01***	***0.04***	***0.92***	***0.32***
Practice of work					
Facility based	4.01	3.74	3.78	3.49	3.83
Community outreach	3.91	3.73	3.85	3.92	4.02
Community home visits	4.10	3.77	3.84	3.70	3.83
Community outreach and Home	3.99	3.88	4.02	3.83	4.09
Other	4.01	3.98	3.93	3.49	3.88
*ANOVA/T test p value*	***0.94***	***0.42***	***0.21***	***0.00***	***0.08***
Years worked in facility					
Less than 5 years	3.95	3.73	3.79	3.55	3.83
5–10 years	4.07	3.80	3.81	3.55	3.87
11–15 years	4.21	3.97	3.96	3.75	4.16
16–20 year	4.12	4.00	3.83	3.66	4.08
Above 20 years	4.33	3.87	3.78	3.18	3.90
*ANOVA/T test p value*	***0.30***	***0.39***	***0.87***	***0.71***	***0.19***
Patient load					
Less than 20 patients	4.00	3.73	3.79	3.55	3.84
20–40 patients	4.02	3.81	3.85	3.60	3.92
41–60 patients	3.87	3.73	3.75	3.51	3.82
61–80 patients	3.41	3.04	3.23	3.21	3.45
More than 80 patients	4.30	4.08	4.10	3.66	3.97
*ANOVA/T test p value*	***0.02***	***0.00***	***0.01***	***0.48***	***0.16***
Licensure					
Less than 5 years	3.89	3.66	3.73	3.54	3.81
5–10 years	4.10	3.82	3.84	3.54	3.91
11–15 years	4.24	3.97	4.05	3.78	4.06
16–20 year	3.99	3.94	3.95	3.65	3.98
Above 20 years	4.01	3.86	3.96	3.60	3.92
*ANOVA/T test p value*	***0.35***	***0.03***	***0.05***	***0.60***	***0.19***
Daily workload					
8 h and less	4.07	3.80	3.84	3.60	3.91
More than 8 h	4.11	3.96	4.10	3.84	4.13
*ANOVA/T test p value*	***0.69***	***0.07***	***0.00***	***0.01***	***0.00***

*Source* Authors computation based on field data

Bold Values are Anova/Test *p*-values

### Determinants of use of evidence by frontline MNCH/RCH staff

In this section, the determinants of use of evidence in practice decisions by frontline MNCH/RCH staff was examined. Using the structural model (i.e. after establishing the convergence and discriminant validity), a stepwise structural regression model was used to examine the association between attitude, access, knowledge and organizational structure on the use of evidence by frontline MNCH/RCH staff in their practice decisions as per Table 8. The results in Table 8 suggest that Attitude has a significant positive effect on the use of evidence and explains 24% of the total variance. In the second step, access was introduced, and the level of variance explained by the model increased by about 5 percentage points, with access also positively affecting the use of evidence. The third and fourth step results suggest that the introduction of the knowledge and organizational structure constructs significantly increases the proportion of variance explained to about 47% and 52% respectively, thus, shedding off the influence of the access variable. The complete model suggest that access is insignificantly associated with the use of evidence when knowledge and organizational structure are controlled for.

**Table 8:** Regression model for main variables

Variables	Model 1	Model 2	Model 3	Model 4
Attitude	0.491***	0.272***	0.143**	0.123**
	(10.196)	(4.87)	(2.924)	(2.534)
Access		0.318***	0.05	0.042
		(6.228)	(0.982)	(0.877)
Knowledge			0.556***	0.393***
			(10.446)	(7.19)
Organisational structure				0.304***
				(5.274)
R Square	0.241	0.293	0.466	0.526
Sample size	492	492	492	492

Dependent variable: Use of evidence

Note also that ***p < 0.01, **p < 0.05 and *p < 0.1. Values in parenthesis represent *t* statistics

In Table 9, other control variables (facility ownership, age, gender, education level, location, years of work and unit/area of work) were introduced, resulting in marginal changes in the *R*^2^ and the coefficients of the key independent variables (attitude, access, knowledge and organizational structure). The results of the additional controls shows that the use of evidence is significantly higher among workers in mission health facilities, but lower in private facilities compared to government facilities. Additionally, the use of evidence is significantly higher among males compared to females and lower among staff who work in a post-natal clinic compared to those in ANC, child welfare clinic and the delivery unit. Also, the length of time in terms of years worked in the health facility is also significantly positively correlated with the use of evidence by frontline MNCH/RCH staff in their practice decisions.

**Table 9:** Regression model including socio-demographic characteristics as control variables

Variables	Model1	Model 2	Model 3	Model 4	Model 5	Model 6	Model 7
Attitude	0.131***	0.13***	0.132***	0.131***	0.132***	0.131***	0.135***
	(2.562)	(2.641)	(2.527)	(2.767)	(2.449)	(2.522)	(2.664)
Access	0.023	0.024	0.018	0.016	0.016	0.011	0.011
	(0.461)	(0.496)	(0.377)	(0.302)	(0.345)	(0.21)	(0.223)
Knowledge	0.384***	0.384***	0.384***	0.382***	0.381***	0.384***	0.37***
	(6.913)	(7.229)	(7.174)	(6.918)	(7.332)	(6.951)	(6.656)
Organisational structure	0.318***	0.318***	0.322***	0.321***	0.32***	0.32***	0.321***
	(5.577)	(5.627)	(5.509)	(5.861)	(5.16)	(5.141)	(5.408)
Facility ownership							
Private facility	− 0.074***	− 0.075***	− 0.08***	− 0.09***	− 0.09***	− 0.087***	− 0.094***
	(2.316)	(2.378)	(2.541)	(2.766)	(2.774)	(2.735)	(2.823)
Mission facility	0.075***	0.075***	0.077***	0.079***	0.079***	0.08***	0.082***
	(2.59)	(2.748)	(2.572)	(2.673)	(2.784)	(2.764)	(2.788)
Quasi facility	0.009	0.008	0.006	0.01	0.01	0.013	0.02
	(0.313)	(0.271)	(0.202)	(0.314)	(0.329)	(0.421)	(0.624)
Urban		0.008	0.022	0.019	0.02	0.021	0.041
		(0.354)	(0.981)	(0.814)	(0.864)	(0.899)	(1.601)
Male			0.062***	0.062***	0.063***	0.065***	0.062***
			(2.498)	(2.581)	(2.447)	(2.631)	(2.631)
Age				0.039*	0.04*	0.03	0.034
				(1.67)	(1.643)	(0.534)	(0.642)
Degree Level and Above					− 0.007	− 0.011	− 0.004
					(0.167)	(0.021)	(0.098)
Years worked in facility						0.055*	0.059*
						(1.778)	(1.747)
Postnatal Clinic							− 0.057**
							(1.657)
*R*^2^	0.542	0.542	0.545	0.546	0.549	0.549	0.553
Sample size	333	333	333	333	333	333	333

*Source* Authors calculation based on field data. Note also that ***p < 0.01, **p < 0.05 and *p < 0.1. The reference category for facility ownership is government facility, urban is rural, male is female and degree level and above is non-degree respectively. Values in parenthesis represent t statistics. Unit assigned to was recoded as binary where all the others (ANC, CWC, and Delivery) were put together as the reference and PNC left in the equation

## Discussion of findings

The study sought to examine the extent of use of evidence by frontline MNCH/RCH staff for practice decisions, as well as the factors that influence the use of such evidence. From the findings, the use of evidence for practice decisions had a score of 3.89 out a total of 5, with the use of both manual and online practice guidelines, protocols and policies as key sources of evidence. In terms of the factors that affect the use of evidence by frontline MNCH/RCH staff, attitudes of staff towards the use of evidence had the highest score (3.99), with organizational structure and processes to facilitate the use of evidence having the lowest score (3.57). The regression results further suggest that frontline MNCH/RCH staff’s attitude towards evidence-based practice, access to relevant information, knowledge of evidence-based practices in MNCH/RCH service provision and organizational structure and processes have a significant positive effect on the use of evidence by frontline MNCH/RCH staff. However, when socio-demographic characteristics of respondents (facility ownership, age, gender, education level, location, years of work and specialty of work) were introduced into the model, access to information remained positive but insignificant. Additionally, the results showed that an MNCH/RCH staff from a mission health facility, being a male and having worked for a longer time was significantly positively correlated with the use evidence for practice decisions.

The overall score of 3.89 out of a total of 5 for use of evidence by MNCH/RCH staff for practice decisions suggest that there is a high level of use of evidence by MNCH/RCH staff, although it can be improved. As expected, evidence seems to be dominated mostly by the use of practice guidelines, protocols and policies. There may be the need to improve the skills of staff to also search for synthesized findings of academic and operations research and use that to inform practice decisions. Within the literature, the use of research evidence (academic or operations research) does not seem to be as popular as practice guidelines, protocols and policies, given the complexities associated with the use of research-based evidence. Issues that have been raised include timeliness and relevance of research evidence to specific practice challenges, relationship between producers and users of evidence and the ability of users to adapt research-based evidence to their context [5, 20]. This may explain the reliance on practice guidelines, protocols and policies.

The results of the study resonate with findings from other jurisdictions (see systematic review by Humphries et al. [20]). Positive Attitudes of healthcare staff such as dieticians and therapist have been suggested as key inputs into evidenced-based practice decision making [18]. There are also examples of physicians, occupational therapist and physical therapist whose positive attitudes towards the use of evidence has actually influenced the use of evidence in their practice [25–28]. It is important also to emphasise that there are instances where positive attitude of healthcare staff has not actually resulted in substantial use of evidence to inform practice decisions [25]. The attitudes of healthcare staff towards the use of evidence is often informed by their perception of what is referred to as evidence [20] and is related to who is producing the evidence and whether it can readily be used in the practice of the healthcare staff [29–31].

The findings also suggest that practice-relevant research evidence is available and accessible to MNCH/RCH staff to some degree. Such availability and accessibility create opportunities for the implementation of strategies that can help to improve MNCH/RCH staff’s understanding of what constitute practice evidence, and consequently ensure that research findings take into consideration the reality of practice. This can be key in improving access to relevant information for evidence-based practice decisions. The difficulty in having access to relevant information for decision-making has also been identified in the literature as a barrier to the use of evidence [30, 32–34]. For example, issues of information overload, the time it takes for research information to be converted into a form that can be used by healthcare workers have been agued in the literature as constituting barriers to the use of evidence [28, 35, 36].

The findings also suggest that knowledge of evidence-based practices in MNCH/RCH is key to using evidence in practice decisions. However, apart from the confidence of staff in sharing and disseminating information with or to their colleagues, all the other indicators on knowledge of evidence-based practices by the MNCH/RCH staff interviewed had relatively lower scores and will therefore need some improvements. Effort to improve knowledge will ensure that staff have the requisite skill and experience needed to utilize available evidence to inform practice decisions. The existing literature suggest that healthcare workers have shortfalls in skills and experience especially in areas of research literacy and research utilization [29, 32, 37]. It is for example suggested that a weaker link that constrain the ability of healthcare workers to utilize evidence in practice decisions include low capacity to; acquire research, assess the reliability, quality, relevance and applicability of research findings and finally ability to summarize research evidence in a manner that can easily be used to inform practice decisions [31].

The results under organizational structure suggest that only 2 (i.e. basic values of continuous learning and a climate of openness, respect and trust among staff) out of the 14 indicators retained in the organizational structure constructs had higher scores. Although the scores of the other 12 indicators were not extremely low, the key message is that the other 12 indicators need some improvement. This will be essential in ensuring that the use of evidence to inform practice decisions among MNCH/RCH staff sees a significant positive change. For example, issues of research leadership, strong professional leadership that facilitate research, putting in place systems that encourages external learning and benchmarking and communication of key research priorities and strategies are essential in this direction. Prior studies have argued that structural rigidities in organizations constitute key constraints to the use of evidence in decision-making [20]. These rigidities manifest in constraints such as low numbers and skill of the required human resources [20, 34, 38], inadequate financial resources [29, 33, 35], workload issues and competing priorities [38], lack of organizational data and systems [34–36], poor senior management support for evidence-informed decision-making [30, 38], poor formal planning and intra-organisational communication [37, 38] and organizational processes that constraints evidence-informed decision-making [32, 37].

## Conclusion

The study sought to examine the level of use of evidence, as well as factors that influence the use of evidence by frontline MNCH/RCH staff in their practice decisions. The results of the study suggest that the level of use of evidence (health policies, guidelines, protocols as well as evidence from academic and facility-based operations research) by MNCH/RCH staff to inform practice decisions is high (3.98 out of 5). In addition, attitude, knowledge, organisational structure, health facility ownership, gender, unit/area of work and years of work experience of individual employees were also found to be correlated with the use of evidence by MNCH/RCH staff for practice decisions.

Notwithstanding the results, it is unlikely that policy makers will seek to change the ownership of health facilities, gender and unit/area of work of individuals in the MNCH/RCH unit of health facilities in other to sustain and improve the use of evidence to inform decisions by frontline MNCH/RCH staff. It will therefore be important that existing and new capacity building programmes that seeks to sustain and improve the use of evidence by frontline health staff emphasise attitudes, knowledge and organisational structure related constraints. It will also be important to ensure that factors such as years of work experience and area/unit where an individual MNCH/RCH staff works is taken into consideration when selecting staff for capacity improvement in relation to the use of evidence. Given that the result of the study shows that organisational structure and access to relevant evidence are the weakest link among the three key determinants, it will be important that management focuses its attention on improving structural challenges within the health facility in general and the MNCH/RCH unit in particular. Indeed, the issues captured under organisational structure (see Table 6) when improved can have implications for attitudes, knowledge and even access and consequently improvement in the use of evidence in practice decisions by MNCH/RCH staff.

The use of evidence to inform practice decisions has long been articulated in the literature [39, 40] as a pathway to strengthening health systems and improving health and healthcare outcomes such as MNCH/RCH outcomes. Although Ghana has over the last couple of years recorded tremendous improvements in health and healthcare outcomes, especially in areas related to MNCH/RCH, existing evidence suggest that Ghana did not achieve MDG goals 4 and 5 and is currently not close to achieving the SDG goals related to MNCH/RC. Thus, addressing key challenges such as access to evidence, knowledge on available evidence and most importantly, systemic challenges that constrain the use of evidence in MNCH/RC practice decisions, will go a long way to improve the use of evidence in practice decisions and consequently, improvement in MNCH/RCH outcomes.

It is also important to acknowledge that a key gap in the design of this study is the exclusion of measurement of capacity of frontline MNCH/RCH to use evidence to inform practice decision. This can be a key area of focus for future studies.

## Supplementary Information

**Additional file 1. **Survey questionnaire for health personnel directly involved in the provision of maternal, newborn and child health (MNCH)/reproductive & child health (RCH) services

## Data Availability

Detailed data based on which the current paper was written is captured in the original report which was submitted to West African Health Organisation (WAHO). The raw data that was collected from the field and formed the basis of the original report is also the property of WAHO. Both the original report and the raw data can be made available upon request from WAHO.
