# Supplementary material for: Determinants of evidence use by frontline maternal, newborn and child health staff in selected health facilities in Ghana
Source: Health Res Policy Syst. 2022 Jun 28;20:77. doi: 10.1186/s12961-022-00881-8 (PMC9238001; doi:10.1186/s12961-022-00881-8)
Supplement: Supplementary file 1 — Additional file 1. Survey questionnaire for health personnel directly involved in the provision of maternal, newborn and child health (MNCH)/reproductive & child health (RCH) services [file 12961_2022_881_MOESM1_ESM.docx]

**QUESTIONNAIRE ID NUMBER: ____ ____ ____**

**SURVEY QUESTIONNAIRE FOR HEALTH PERSONNEL DIRECTLY INVOLVED IN THE PROVISION OF MATERNAL, NEWBORN AND CHILD HEALTH (MNCH)/REPRODUCTIVE & CHILD HEALTH (RCH) SERVICES**

***The following questions are designed to help us determine your capacity for evidence-based policy making so as to serve as the basis for future research works in promoting the use of research evidence for in the provision of MNCH/RCH services in the health sector.***

**Please answer the questions as correctly as possible. The questionnaire is purely for research purpose only.**

**Date**________________________________

**SECTION ONE: ORGANISATIONAL INFORMATION**

***(Circle only one option)***

1. **Facility type :** CHPS compound…..1 Health centre……2 Private clinic……3 Poly Clinic…….4

Maternity Home…...5 District hospital….6 Private hospital…7

Mission hospital……8 Quasi hospital....…9 Other(specify)_____________________10

1. **Facility Ownership** Private facility………….1 Government facility……2 Mission facility……….3

Other(specify)__________________________________4

1. **Community name_____________________________**
2. **Type of Location** Urban………..1 Rural………..2
3. **District name _______________________________**
4. **Region name ________________________________**

**SECTION TWO: BIODATA OF RESPONDENTS**

***(Circle only one option)***

1. **Sex** Male……1 Female…….2
2. **Age** ___ ___
3. **Highest Education level completed**: Certificate…….1 Diploma………2 First Degree………3

Masters( MA, MPH, MSC, MPhil, etc)….4 PhD…….5

Other (specify)_____________________________6

1. **Years practices as a health personnel/licensed ___ ____ *(please input 00 if less than a year)***
2. **Current/usual assigned service** ANC…..1 PNC……2 Child Welfare clinic……..3

Delivery……...4 Other(specify)__________________5

Other(specify)_________________6  ***[multiple answers allowed]***

1. **Current department ________________________________**
2. **Specific unit (eg: Emergency, OPD, labour ward, etc)________________________**
3. **Current/usual commodity by life stage in MNCH/RCH service provision**

Reproductive health……1 Maternal health……2 Child health……3 New born health………4

Other(specify)_________________________5 Other(specify)_________________________6

1. **Usual area/location of practice/work** Facility-based……..1 Community-based……...2

Other(specify)____________________________3

1. **Years worked in this facility ___ ___ *(Please, input 00 if less than a year)***
2. **Professional Affiliation _____________________________________**
3. **Position/Designation in this facility _______________________________________**
4. Average daily patient load at primary work:____ ____
5. **Work hours per week: ___ ___ ___**
6. **Provides patient care outside of regular work hours**: Yes…..1 No…..2 Sometimes……3

Occasionally….4 Not at all……5

1. **Religious Affiliation:** 01=No Religion 02=Catholic 03=Protestant (Presby,Methodist,Lutheran)

04=Pentecostal/Charismatic 05=Other Christian 06=Islam 07=Traditional/Spiritualist

96=Other (Specify)________________________

1. **Years of professional licensure**: ____ _____ years
2. **Member of professional specialist organization(s)/fellowships:** (GCPS, GCNM, etc) 1.__________________________________________________________

2.__________________________________________________________

3.__________________________________________________________

**SECTION THREE**

**2. Personal attitudes, access to information, knowledge of EBP, organisational structure and process and the use of Evidence for Decision Making**:

There are a number of reasons that may constitute a influence your use of evidence in MNCH/RCH service provision decision making. To the best of your ability, please rate your level of agreement to the listed statements on the scale of **1 (strongly disagree) to 5 (strongly agree). This is** to **describe your point of view towards your use of Evidence in MNCH/RCH** in your work as a frontliner in MNCH/RCH service provision.

Please tick the appropriate response that reflects you.

***Strongly disagree* – 1 *Disagree* – 2  *Indifferent* -3 *Agree* - 4 *Strongly agree* – 5**

| **ATTITUDE towards the use of evidence-based practice in MNCH/RCH service provision.** | | **1** | **2** | **3** | **4** | **5** |
| --- | --- | --- | --- | --- | --- | --- |
| 1. | My workload is not too great for me to keep up to date with all the new evidence. |  |  |  |  |  |
| 2. | The adaptation of evidence-based practice does not place unreasonable demand on my practice time. |  |  |  |  |  |
| 3. | I am ok if my clinical practice is questioned |  |  |  |  |  |
| 4. | My use of evidence in my practice does not wastes my time. |  |  |  |  |  |
| 5. | I like changing to something new than rather stick to tried and trusted methods. |  |  |  |  |  |
| 6. | Application of evidence-based practice is necessary in my practice. |  |  |  |  |  |
| 7. | Literature and research findings are necessary in my day-to-day practice. |  |  |  |  |  |
| 8. | I am interested in learning or the skills necessary to incorporated evidence-based practice in my daily practice. |  |  |  |  |  |

Please tick the appropriate response that reflects you.

***Strongly disagree* – 1 *Disagree* – 2  *Indifferent* -3 *Agree* - 4 *Strongly agree* – 5**

| **ACCESS TO INFORMATION towards the use of evidence-based practice in MNCH/RCH service provision.** | | **1** | **2** | **3** | **4** | **5** |
| --- | --- | --- | --- | --- | --- | --- |
| 1. | Available research evidence is relevant to my practice. |  |  |  |  |  |
| 2. | I have sufficient understanding of what constitutes evidence in my practice. |  |  |  |  |  |
| 3. | I have confidence in findings from research. |  |  |  |  |  |
| 4. | I have some degree of access to information related to my practice. |  |  |  |  |  |
| 5. | Research findings often take into consideration the complex reality of my practice |  |  |  |  |  |
| 6. | It does not take too long to produce research and release findings. |  |  |  |  |  |
| 7. | There is so much available research (information) that I know those to select for my practice. |  |  |  |  |  |

Please tick the appropriate response that reflects you.

***Strongly disagree* – 1 *Disagree* – 2  *Indifferent* -3 *Agree* - 4 *Strongly agree* – 5**

| **KNOWLEDGE of evidence-based practice in MNCH/RCH service provision.** | | **1** | **2** | **3** | **4** | **5** |
| --- | --- | --- | --- | --- | --- | --- |
| 1. | I have very good research skills. |  |  |  |  |  |
| 2. | I have what can be considered to be above average in information technology skills. |  |  |  |  |  |
| 3. | I have above average in the skills for monitoring and reviewing practice skills. |  |  |  |  |  |
| 4. | I have above average skills to convert my information needs into a research question. |  |  |  |  |  |
| 5. | I am aware of major sources of information for which I can extract evidence for the purposes of my practice. |  |  |  |  |  |
| 6. | I am very much capable of identifying gaps in my professional practice. |  |  |  |  |  |
| 7. | I have enough knowledge to be able to extract evidence from available sources for my practice. |  |  |  |  |  |
| 8. | I have enough ability to analyse critically, evidence against set standards for my practice. |  |  |  |  |  |
| 9. | I have enough ability to determine how valid (close to the truth) an evidence material is for my practice. |  |  |  |  |  |
| 10. | I have enough ability to determine how useful (clinically applicable) an evidence material is for my practice. |  |  |  |  |  |
| 11. | I have enough ability to apply information to individual cases. |  |  |  |  |  |
| 12. | I am confident in my ability to share ideas and information with colleagues. |  |  |  |  |  |
| 13. | I have the confidence in disseminating new ideas about care to colleagues. |  |  |  |  |  |
| 14. | I have enough ability to review my own practice. |  |  |  |  |  |

Please tick the appropriate response that reflects you.

***Strongly disagree* – 1 *Disagree* – 2  *Indifferent* -3 *Agree* - 4 *Strongly agree* – 5**

| **ORGANISATIONAL STRUCTURE AND PROCESS in evidence-based practice in MNCH/RCH service provision.** | | 1 | 2 | 3 | 4 | 5 |
| --- | --- | --- | --- | --- | --- | --- |
| 1. | There is enough time to use evidence in decision making at my workplace. |  |  |  |  |  |
| 2. | My workplace provides the necessary research resources for use of evidence in MNCH/RCH provision. |  |  |  |  |  |
| 3. | There are enough human resources at my workplace that promote use of evidence for my practice. |  |  |  |  |  |
| 4. | There are enough financial resources at my workplace that promote use of evidence for my practice. |  |  |  |  |  |
| 5. | My workplace provides the necessary data and systems to apply EBP. |  |  |  |  |  |
| 6. | Management supports the use of current research in practice. |  |  |  |  |  |
| 7. | My work place promotes the practice of outward looking and the sharing of resources and information to support the use of evidence. |  |  |  |  |  |
| 8. | There is specific infrastructure within the organisation to support the research process. |  |  |  |  |  |
| 9. | Open communication is a characteristic of my workplace. |  |  |  |  |  |
| 10. | We have an environment that supports the application of EBP. |  |  |  |  |  |
| 11. | We have a system that promotes external contacts and allows us to learn of successful practices from other organisations. |  |  |  |  |  |
| 12. | The environment at my work place embraces change in support of the use of evidence in my practice. |  |  |  |  |  |
| 13. | The facility promotes a climate of openness, respect and trust among all levels of professionals. |  |  |  |  |  |
| 14. | The basic values of the Department include continuous learning as a key to improvement in client care. |  |  |  |  |  |
| 15. | Managers frequently involve staff in important decisions relating to clinical care procedures, protocols and guidelines. |  |  |  |  |  |
| 16. | Managing knowledge is central to the organisation's strategy. |  |  |  |  |  |
| 17. | Management clearly communicates key research strategy and priorities |  |  |  |  |  |
| 18. | There is widespread support and acceptance of the organisation's mission statement |  |  |  |  |  |
| 19. | There is a strong professional leadership within the organisation that facilitates research |  |  |  |  |  |
| 20. | There is the existence of committees and representation in all professionals on research committee, councils etc |  |  |  |  |  |
| 21. | Professionals are encouraged to question their practices. |  |  |  |  |  |
| 22. | Problems are discussed openly and analytically to learn from experiences and without blame. |  |  |  |  |  |
| 23. | There are best practice repositories in my organisation, recognising and valuing existing knowledge |  |  |  |  |  |
| 24. | Attendance at conferences/presentations that give information on research and exposure to new information is encouraged. |  |  |  |  |  |
| 25. | Professionals are encouraged to discuss experiences/expertise with colleagues in regular meetings. |  |  |  |  |  |
| 26. | Technology to support collaboration is available and made available to professionals to promote research |  |  |  |  |  |
| 27. | Multi-professional review and audit teams support teamwork |  |  |  |  |  |
| 28. | We are encouraged to attend training programmes to develop expertise with funding from the facility. |  |  |  |  |  |

Please tick the appropriate response that reflects you.

***Strongly disagree* – 1 *Disagree* – 2  *Indifferent* -3 *Agree* - 4 *Strongly agree* – 5**

| **USE OF EVIDENCE in MNCH/RCH service provision.** | | **1** | **2** | **3** | **4** | **5** |
| --- | --- | --- | --- | --- | --- | --- |
| 1. | I often formulate a clearly answerable question as the beginning of the process towards filling a gap. |  |  |  |  |  |
| 2. | I often track down the relevant evidence once I have formulated a question. |  |  |  |  |  |
| 3. | I often critically appraise, against set criteria, any literature I discover. |  |  |  |  |  |
| 4. | I often integrate the evidence I find with my expertise into my practice. |  |  |  |  |  |
| 5. | I often evaluate the outcomes of my practice. |  |  |  |  |  |
| 6. | I often share this information with colleagues. |  |  |  |  |  |
| 7. | I actively seek practice guidelines available for my practice. |  |  |  |  |  |
| 8. | I use practice guidelines in my practice. |  |  |  |  |  |
| 9. | I am aware that practice guidelines are available online. |  |  |  |  |  |
| 10. | I am able to access the practice guidelines online. |  |  |  |  |  |

| 11. | Understanding and Use of Clinical Practice Guidelines. |  |  |  |  |  |
| --- | --- | --- | --- | --- | --- | --- |
| 12. | I use professional literature and research findings in the process of clinical  decision making. |  |  |  |  |  |
| 13. | I use practice protocols, policies and guidelines in my practice |  |  |  |  |  |
| 14. | I am able to incorporate patient preferences with practice protocols and guidelines |  |  |  |  |  |
| 15. | I actively seek practice guidelines and protocols pertaining to areas of my practice |  |  |  |  |  |
| 16. | I am confident in my ability to find relevant literature to answer clinical questions |  |  |  |  |  |
| 17. | I am familiar with the current policies and guidelines related to my line of practice *(e.g. Reproductive Health Protocol,2016; New borne Strategy, 2014*) |  |  |  |  |  |
| 18. | I make patient care decisions Based on Economic Evaluation |  |  |  |  |  |
| 19. | I incorporate patient preferences and values in evidence based decision making |  |  |  |  |  |
| 20. | I have the ability to explain procedures and management practices to the understanding of my patients |  |  |  |  |  |

Please tick the appropriate response that reflects you.

***Strongly disagree* – 1 *Disagree* – 2  *Indifferent* -3 *Agree* - 4 *Strongly agree* – 5**

|  | **PERCEIVED BENEFITS AND LIMITATION in use of EBP in MNCH/RCH service provision** | **1** | **2** | **3** | **4** | **5** |
| --- | --- | --- | --- | --- | --- | --- |
| 1. | Literature and research findings help improve patient care. |  |  |  |  |  |
| 2. | EBP helps me make decision about patient care. |  |  |  |  |  |
| 3. | Literature and research findings are useful in my day-to-day practice. |  |  |  |  |  |
| 6. | I have adequate access to evidence based policies, protocols and guidelines. |  |  |  |  |  |
| 8. | I am able to apply research findings in my patient population. |  |  |  |  |  |
| 11. | EBP takes into account patient preferences. |  |  |  |  |  |
| 12. | EBP takes into account the limitations of my clinical practice setting. |  |  |  |  |  |
| 13. | EBP places no demand on medicine nursing and midwifery. |  |  |  |  |  |

***Thank you for your participation***
